# Development of Neurogenic Detrusor Overactivity after Thoracic Spinal Cord Injury Is Accompanied by Time-Dependent Changes in Lumbosacral Expression of Axonal Growth Regulators

**DOI:** 10.3390/ijms23158667

**Published:** 2022-08-04

**Authors:** Sílvia Sousa Chambel, Ana Ferreira, Raquel Oliveira, Rafael Miranda, Luís Vale, Carlos Reguenga, Martin E. Schwab, Célia Duarte Cruz

**Affiliations:** 1 Experimental Biology Unit, Department of Biomedicine, Faculty of Medicine of Porto, University of Porto, 4200-319 Porto, Portugal; 2 Translational NeuroUrology, Instituto de Investigação e Inovação em Saúde-i3S, Universidade do Porto, 4200-135 Porto, Portugal; 3 Pain Neurobiology, Instituto de Investigação e Inovação em Saúde-i3S, Universidade do Porto, 4200-135 Porto, Portugal; 4 Departament of Urology, Centro Hospitalar S. João, 4200-319 Porto, Portugal; 5 Pharmacology and Therapeutics Unit, Department of Biomedicine, Faculty of Medicine of Porto, University of Porto, 4200-319 Porto, Portugal; 6 Institute for Regenerative Medicine, University of Zurich, Wagistrasse 27, 8952 Schlieren, Switzerland

**Keywords:** spinal cord injury, repulsive molecules, axonal growth, bladder dysfunction, neurogenic detrusor overactivity

## Abstract

Thoracic spinal cord injury (SCI) results in urinary dysfunction, which majorly affects the quality of life of SCI patients. Abnormal sprouting of lumbosacral bladder afferents plays a crucial role in this condition. Underlying mechanisms may include changes in expression of regulators of axonal growth, including chondroitin sulphate proteoglycans (CSPGs), myelin-associated inhibitors (MAIs) and repulsive guidance molecules, known to be upregulated at the injury site post SCI. Here, we confirmed lumbosacral upregulation of the growth-associated protein GAP43 in SCI animals with bladder dysfunction, indicating the occurrence of axonal sprouting. Neurocan and Phosphacan (CSPGs), as well as Nogo-A (MAI), at the same spinal segments were upregulated 7 days post injury (dpi) but returned to baseline values 28 dpi. In turn, qPCR analysis of the mRNA levels for receptors of those repulsive molecules in dorsal root ganglia (DRG) neurons showed a time-dependent decrease in receptor expression. In vitro assays with DRG neurons from SCI rats demonstrated that exposure to high levels of NGF downregulated the expression of some, but not all, receptors for those regulators of axonal growth. The present results, therefore, show significant molecular changes at the lumbosacral cord and DRGs after thoracic lesion, likely critically involved in neuroplastic events leading to urinary impairment.

## 1. Introduction

Normal body function requires an intact spinal cord, functioning as a relay and processing centre for peripherally generated input and conveying supraspinal commands to effector organs. The spinal cord is complex and comprises neurons, glial cells, and ascending and descending nerve fibres. Spaces between cells and fibres are occupied by extracellular matrix (ECM), a scaffold composed of secreted complex proteins and sugars, required to support cellular activity and survival [1]. Traumatic spinal cord injury (SCI) significantly alters the dynamics and composition of ECM at the lesion site as a consequence of neuronal and glial cell death, activation of surviving cells and immigration of peripheral inflammatory cells.

Post-SCI changes in ECM composition at the peri-lesion site reflect accumulation of inhibitory cues that participate in axon guidance and regulate axonal growth. These molecules are normally present at low levels, but some are overproduced in response to injury, creating a highly repulsive environment at the lesion site and impairing repair of disrupted neuronal tracts [2,3]. Sources of these inhibitory molecules include astrocytes, oligodendrocytes and the myelin sheath. Binding of these proteins to their receptors, present in neurons, causes actin depolymerization, growth cone collapse and blockade of regenerative growth after SCI [4]. The most well-studied repulsive molecules fall into three major categories: chondroitin sulphate proteoglycans (CSPGs), myelin-associated inhibitory proteins (MAIs) and repulsive guidance molecules.

Astrocyte-derived chondroitin sulphate proteoglycans (CSPGs) are glycoproteins that contribute to the structural scaffold of the spinal ECM and have been associated with regulating synaptic stability, reduced plasticity and inhibition of axonal elongation [5,6]. Examples of CSPGs include Neurocan and Phosphacan [7]. CSPG-mediated axonal growth cone collapse is linked with the activation of receptor-type protein tyrosine phosphatase sigma (RPTPσ) [8], leukocyte common antigen-related phosphatase (LAR) [9], Nogo receptor (NgR1) and Nogo receptor 3 (NgR3) [10].

Myelin-associated inhibitory proteins (MAIs) include Nogo-A [11], myelin-associated glycoprotein (MAG) [12] and oligodendrocyte-myelin glycoprotein (OMgp) [13,14]. Nogo-A is the most well-described MAI. It is produced mostly by oligodendrocytes [15,16], but also by certain subpopulations of central and peripheral neurons [11,17]. Nogo-A signals with two binding regions through two receptor components, NgR1 [18], forming a receptor complex with leucine rich repeat and immunoglobulin-like domain-containing NgR-interacting protein 1 (LINGO-1) [19] and p75 [20], and/or TROY [21,22], and sphingosine 1-phosphate receptor 2 (S1PR2) [23]. Nogo-A has been associated with regulation of neuroplasticity, learning and memory [24,25,26]. MAG is involved in maintenance of myelin sheaths. It is expressed at high levels by oligodendrocytes in the central nervous system (CNS) while Schwann cells, in the peripheral nervous system, may also produce MAG [27]. MAG can signal through the NgR1/LINGO1/p75 and/or TROY receptor complex [28]. OMgp is present in the cell membrane of oligodendrocytes and the glial–axonal interface of myelinated axons both in the brain and spinal cord [29,30]. OMgp is involved in the inhibition of neurite outgrowth following injury [31,32] and acts via NgR1/LINGO1/p75 and/or TROY receptor complex [13,28].

Guidance molecules, besides playing an important role in neural circuit assembly during development, are also involved in CNS response after injury [33]. Ephrins and Semaphorins are involved in the guidance of ascending sensory and descending motor pathways, but after SCI, exert a highly repulsive effect towards axons [34]. Repulsive Guidance Molecule A (RGMa) is present in myelin and accumulates in the glial scar following a spinal cord injury [35]. After SCI, RGMa is expressed in neurons, oligodendrocytes, astrocytes, activated microglia and macrophages [36], and binds to its neuronal receptor, Neogenin 1 (Neo1), [37], inhibiting neurite outgrowth [38,39].

Changes in peri-lesional ECM have been well studied and linked to impaired recovery of sensorimotor function in SCI patients [4]. However, albeit less studied, there is indication that changes in the ECM composition may also occur in spinal areas distant from the lesion site [40], where they likely contribute to maladaptive plasticity. For example, development of SCI-induced urinary impairment, one of the major degraders of quality of life for SCI patients [41], is associated with abnormal sprouting at the lumbosacral spinal cord of the central processes of sensory peptidergic afferents [42,43,44]. While the fine molecular mechanisms regulating this aberrant axonal growth are still mostly unresolved, it may reflect changes in lumbosacral spinal ECM, making it more permissive to expansion of the central processes of sensory afferents. These ECM modifications remain poorly investigated. Moreover, it is not well understood if sprouting sensory neurons regulate the expression of the receptors for these centrally expressed repulsive molecules. Therefore, this study investigated whether levels of repulsive molecules, including CSPGs (Phosphacan and Neurocan), MAIs (Nogo-A, OMgp and MAG) and RGMa, are altered in the lumbosacral cord of SCI animals submitted to thoracic spinal transection. The expression of receptors for these repulsive cues in sensory neurons was also analysed.

## 2. Results

### 2.1. Spinal Cord Transection Induces Urinary Dysfunction

**Figure 1:**
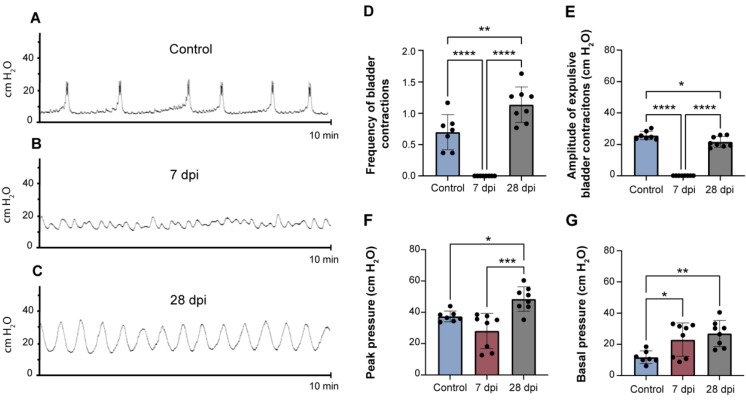
Representative cystometrograms depicting the voiding pattern of animals with intact spines (control) and after spinal cord injury. Control animals exhibited a normal pattern of bladder reflex activity (**A**). SCT animals exhibited hallmarks of bladder dysfunction. At 7 days post-injury (7 dpi), bladder contractions with an amplitude higher than 5 cm H_2_O were nonexistent (**B**), while at 28 days post-injury (28 dpi) a typical pattern of NDO was well established (**C**), evidencing signs of strong, frequent and involuntary bladder contractions. Analysis of urodynamics parameters, such as frequency of bladder contractions (**D**), amplitude of expulsive bladder contractions (**E**) and peak (**F**) and basal pressures (**G**) are also detailed. Graphs represent mean ± SD and *p* < 0.05 was considered statistically significant. * *p* ≤ 0.05; ** *p* ≤ 0.01; *** *p* ≤ 0.001; **** *p* ≤ 0.0001; one-way ANOVA followed by Tukey’s multiple comparison test.

At the end of each time point, cystometries under urethane anaesthesia were performed to evaluate urinary function. Spinal intact animals (control) exhibited a normal pattern of bladder reflex activity (Figure 1A), showing regular contractile activity. The frequency of bladder contractions was 0.7 ± 0.3 contractions/min (Figure 1D). The amplitude of bladder contractions was 25.7 ± 2.5 cm H_2_O (Figure 1E), while the peak pressure was 37.4 ± 3.2 cm H_2_O (Figure 1F) and the basal pressure was 11.7 ± 3.7 cm H_2_O (Figure 1G).

Thoracic spinal transection caused marked alterations in bladder function, observable both at 7 and 28 days post-injury (dpi). While at 7 dpi, bladder contractions were abolished (Figure 1B–E; *p* < 0.001 vs. control animals), at 28 dpi there were evident signs of bladder overactivity (Figure 1C). In this experimental group, the frequency of bladder contractions was 1.1 ± 0.3 contractions/min (Figure 1D). The amplitude of contractions was 21.6 ± 3.4 cm H_2_O (Figure 1E), while peak pressure was 48.5 ± 7.8 cm H_2_O (Figure 1F) and basal pressure was 26.9 ± 8.3 cm H_2_O (Figure 1G). For all parameters, values were significantly different from those observed in control animals and indicate the development of SCI-induced urinary dysfunction.

### 2.2. SCT-Induced Urinary Dysfunction Is Accompanied by Increased Spinal Expression of GAP43

**Figure 2:**
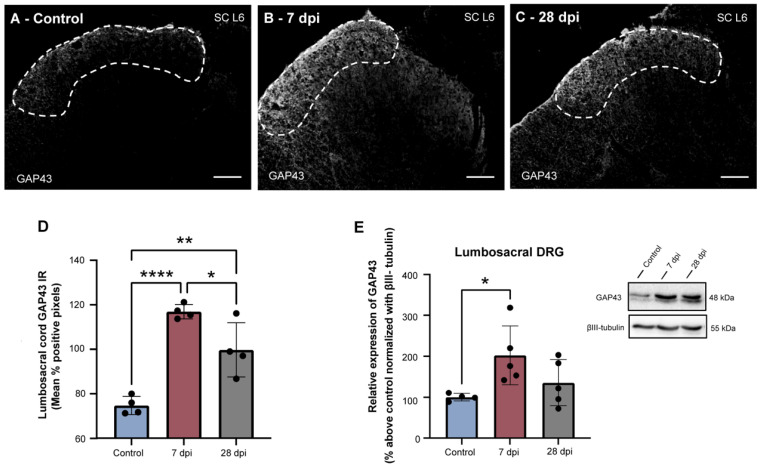
Time-dependent alterations in GAP43 levels at the lumbosacral spinal cord and DRG after spinal cord injury. Distribution of GAP43 in the L6 segment of the lumbosacral spinal cord (**A**–**C**). GAP43 was detected via fluorescent immunohistochemistry in the L5, L6 and S1 spinal segments of control and SCT animals. GAP43 expression was predominantly observed in the superficial dorsal horn (laminae I and II). Levels of spinal GAP43 expression increased significantly 7 dpi (**D**). Twenty-eight days post-injury, GAP43 levels at the lumbosacral spinal cord remained elevated compared to control animals (**D**). In lumbosacral DRG neurons, Western blotting analysis showed a significant increase in GAP43 expression 7 dpi, compared to control animals (**E**). Scale bar: 100 µm. Graphs represent mean ± SD and *p* < 0.05 was considered statistically significant. * *p* ≤ 0.05; ** *p* ≤ 0.01; **** *p* ≤ 0.0001; one-way ANOVA followed by Tukey’s multiple comparison test.

Previous studies assessing animals submitted to complete spinal cord transection have demonstrated that post-SCI urinary impairment courses with axonal sprouting of sensory afferents at the lumbosacral spinal cord [42,45]. To confirm whether the SCI-induced bladder dysfunction observed here was also linked to sprouting of the central processes of sensory afferents, levels of GAP43, an established marker of axonal sprouting [46], were evaluated. GAP43 expression was mostly found in laminae I-II of the lumbosacral spinal cord (L5-S1) (Figure 2), with some processes present in other regions such as the dorsal commissure, corticospinal tract and dorsolateral funiculus in spinal intact animals (not showed). While in control animals, GAP43 immunoreactivity was low (Figure 2A,D), GAP43 expression levels significantly increased 7 dpi (Figure 2B,D; *p* < 0.0001 vs. control animals). At twenty-eight dpi, GAP43 levels decreased (Figure 2C,D; *p* < 0.05 vs. 7 dpi animals) but remained elevated compared to control animals (*p* < 0.01). In the DRG, GAP43 was also significantly elevated at 7 dpi and slightly decreased at 28 dpi (Figure 2E; *p* < 0.05 versus control). These observations confirm that development of urinary impairment coincides with lumbosacral axonal sprouting.

### 2.3. Thoracic SCT Induces Changes in Lumbosacral Expression of Axonal Growth Regulators

While axonal sprouting is very limited in the adult central nervous system [47], spinal lumbosacral GAP43 expression upregulation suggests changes in the extracellular environment, which could have become less repulsive, allowing the expansion of neuronal processes after a thoracic spinal injury. To explore this hypothesis, the expression of proteins known to control axonal growth was analysed. 

**Figure 3:**
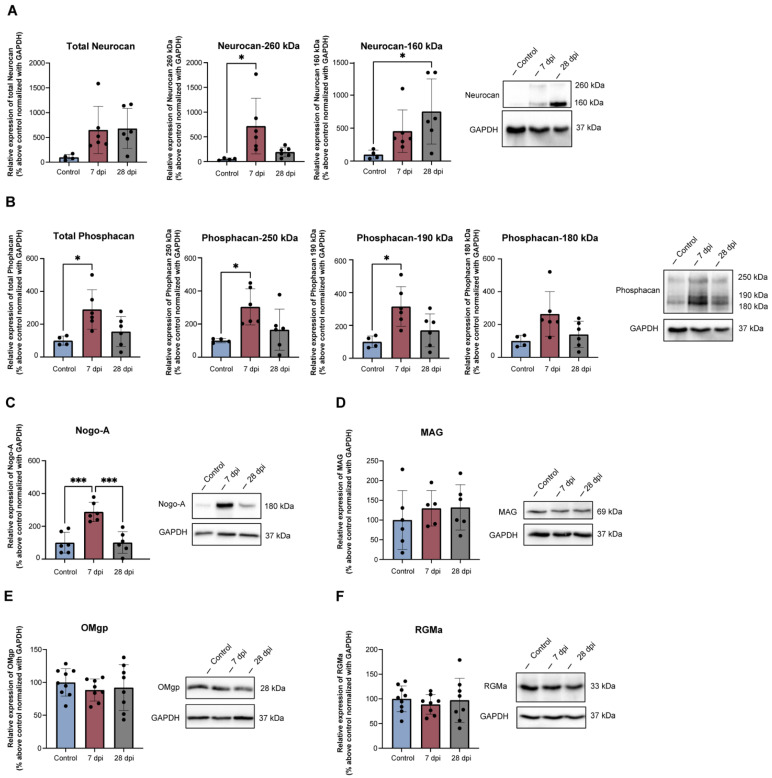
Time-dependent changes in repulsive proteins expression after thoracic spinal cord injury. Western blots of Neurocan (**A**), Phosphacan (**B**), Nogo-A (**C**), MAG (**D**), OMgp (**E**) and RGMa (**F**) and respective ratio against the endogenous protein GAPDH at the lumbosacral spinal cord in spinal intact animals (control) and after SCT. Total Neurocan (A) expression did not show any significant differences between controls and SCT animals, but Neurocan-250 significantly increased 7 dpi and Neurocan-160 increased significantly 28 dpi. Total Phosphacan (**B**) and Nogo-A (**C**) expression significantly increased 7 dpi (days post-injury), decreasing at 28 dpi. Expression of MAG (**D**), OMgp (**E**) and RGMa (**F**) was not changed by the thoracic lesion. Graphs represent mean ± SD and *p* < 0.05 was considered statistically significant. * *p* ≤ 0.05; *** *p* ≤ 0.001; one-way ANOVA followed by Tukey’s multiple comparison test.

Total Neurocan levels detected by Western blot were not significantly changed by SCT (Figure 3A), but changes in levels of specific isoforms were found. Neurocan-260 significantly increased 7 dpi (*p* < 0.05 vs. control animals) and Neurocan-160 significantly increased 28 dpi (*p* < 0.05 vs. control animals) (Figure 3A). Total Phosphacan expression in the lumbosacral cord, as well as Phosphacan-250 kDa and Phosphacan-190 kDa, showed a significant three-fold increase at 7 dpi (Figure 3B). Immunohistochemical analysis of Phosphacan expression at the lumbosacral cord showed similar results (Supplementary Figure S2). Importantly, while Phosphacan and GAP43 were present in the same spinal areas, there was no overlap of immunofluorescence.

The expression of MAIs was also analysed. Lumbosacral expression of Nogo-A was low in spinal intact rats but had a significant three-fold increase 7 dpi (*p* < 0.001 vs. control animals), returning to baseline 28 dpi (*p* < 0.001 vs. 7 dpi animals) (Figure 3C). Like Phosphacan, immunodetection of Nogo-A showed a similar variation (Supplementary Figure S3), with Nogo-A and GAP43 present in the same spinal laminae but without overlap.

For other MAIs, also studied here, no changes were found in levels of MAG (Figure 3D) and OMgp (Figure 3E). Expression of RMGa was also unaltered (Figure 3F). These results demonstrate the occurrence of dynamic patterns of expression of regulators of axonal growth in the lumbosacral cord, restricted to some inhibitory cues.

### 2.4. Alterations in Lumbosacral Expression of Axonal Growth Regulators Is Accompanied by DRG Changes in Levels of Specific Receptors

**Figure 4:**
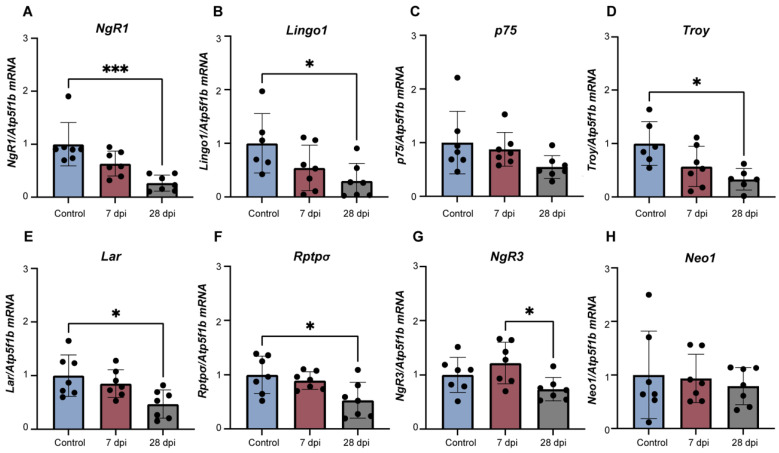
Levels of mRNA of myelin-associated inhibitors (MAI) receptor complex *NgR1* (**A**), *Lingo1* (**B**), *p75* (**C**) and *Troy* (**D**), CSPGs receptors *Lar* (**E**), *Rptpσ* (**F**) and *NgR3* (**G**)*;* and RGMa receptor *Neo1* (**H**) in lumbosacral (L5-S1) DRG of spinal intact (control) and SCT animals. After thoracic spinal cord transection, mRNA levels of receptor complex *NgR1*/*Lingo1*/*p75* (**A**–**C**) and/or *Troy* (**D**) tended to significantly decrease 28 dpi (days post-injury). *Lar*/*Rptpσ*/*NgR3* (**E**–**G**) also followed the same trend. RGMa receptor *Neo1* mRNA levels (**H**) were not changed by the thoracic spinal cord lesion. Graphs represent mean ± SD and *p* < 0.05 was considered statistically significant. * *p* ≤ 0.05; *** *p* ≤ 0.001; one-way ANOVA followed by Tukey’s multiple comparison test.

As GAP43 upregulation in the superficial laminae of the lumbosacral cord, where visceral afferents are known to project [48,49], was inconsistent with changes in CSPGs and MAIs in the lumbosacral cord, we hypothesized that the expression of their specific receptors was altered in lumbosacral sensory afferents, making them insensitive to inhibitory cues and allowing axonal elongation. To investigate this, mRNA levels of receptor complexes were analysed by RT-qPCR for MAIs: *NgR1/Lingo1/p75* and/or *Troy;* CSPGs: *NgR1*/*NgR3*, *Rptpσ*/*Lar;* and RGMa: *Neo1*. Levels of mRNA encoding for MAI complex receptor *NgR1*/*Lingo1/p75* and/or *Troy* (Figure 4A–D) significantly decreased in a time-dependent manner. *NgR1*, *Lingo1* and *Troy* significantly decreased at 28 dpi compared to control animals, while *p75* mRNA levels did not reach statistical significance, supporting the duality of this receptor complex (Figure 4A, *p* < 0.001 vs. control; Figure 4B,D, *p* < 0.05 vs. control).

Likewise, mRNA expression of *NgR3* (Figure 4G), *Rptpσ* (Figure 4F) and *Lar* (Figure 4E), part of the CSPG receptor complexes *NgR1/NgR3* and *Rptpσ*/*Lar*, was also time-dependently reduced at 28 dpi (*NgR3*: Figure 4B, *p* < 0.05 7 dpi vs. 28 dpi animals; *Rptpσ*/*Lar*: Figure 4E,F, *p* < 0.05 control vs. 28 dpi animals). In contrast, mRNA levels of the RGMa receptor *Neo1* were similar between experimental groups (Figure 4H). These results show DRG neurons express receptors for spinal inhibitory cues, the levels of which vary with disease progression.

### 2.5. Rhoa/Rock Module Is Not Altered in Lumbosacral Drg Neurons following Thoracic Spinal Transection

**Figure 5:**
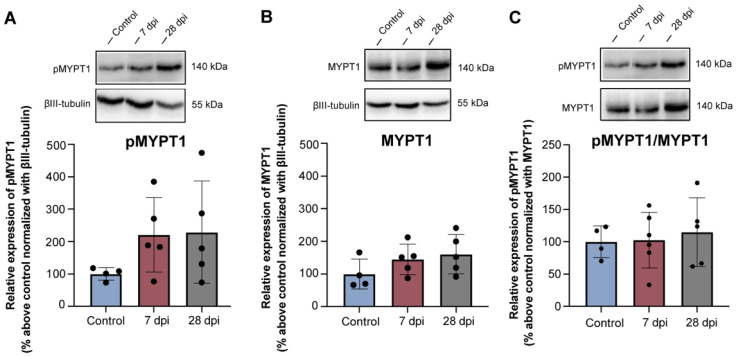
Time-dependent expression of MYPT1 and pMYPT1 in lumbosacral (L5-S1) DRG neurons of spinal intact (control) and SCT (7 and 28 dpi (days post-injury)) animals, determined by Western blotting. pMYPT1 (**A**) and MYPT1 (**B**) were normalized against βIII-tubulin and levels of pMYPT1 were also normalized against endogenous levels of MYPT1 (**C**). In all cases, no statistical differences were found between experimental groups. Graphs represent mean ± SD. One-way ANOVA followed by Tukey’s multiple comparison test.

Binding of MAIs, CSPGs and repulsive guidance molecules to their specific receptors causes collapse of the axonal cone via activation of RhoA and inactivation of protein kinase B (Akt) signalling pathways downstream of the receptors [50]. A reduction in the expression of those receptors would result in lack of activation of RhoA and its effector ROCK, which is responsible for mediating rearrangements of the actomyosin cytoskeleton, consistent with the ongoing axonal sprouting and increased GAP43 expression observed here (Figure 2). Since ROCK is also responsible for the phosphorylation of myosin phosphatase target subunit 1 (MYPT1) at Thr696, inactivating it and leading to neurite retraction and neurite outgrowth inhibition [51], the expression of MYPT1 and phosphorylated MYPT1 (pMYPT1) at Thr696 was resolved by Western blotting in lumbosacral (L5-S1) DRG samples. No changes were found between control and SCT groups in the expression of pMYPT1 (Figure 5A) or MYPT1 (Figure 5B). Ratio between pMYPT1/MYPT1 (Figure 5C) followed the same pattern, indicating lack of activation of the RhoA/ROCk signalling pathway, consistent with ongoing axonal growth.

### 2.6. NGF Regulates the Expression of Receptors for Axonal Guidance Cues in DRG Neurons

To investigate the mechanisms governing the peripheral downregulation of MAIs, CSPGs and repulsive guidance-molecules-specific receptors, L5-S1 DRG neurons were collected from control and SCT animals (7 and 28 days post-injury) and cultured for 22 h in different concentrations of NGF—0, 50 and 100 ng/mL of NGF. NGF was chosen, as it has been shown to be upregulated following SCT in rostral and caudal spinal cord (in relation to the injury site), in the cell body of DRG neurons and in peripheral organs such as the bladder [52,53,54]. NGF is also known as an inducer of axonal elongation [55,56] and its increase has been linked to SCT-induced urinary dysfunction [57,58]. 

**Figure 6:**
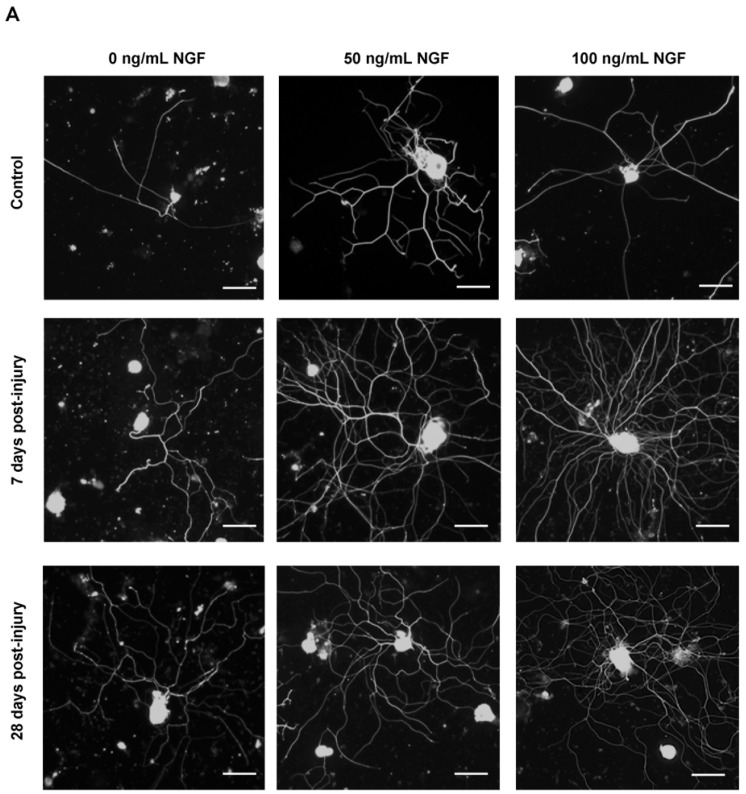

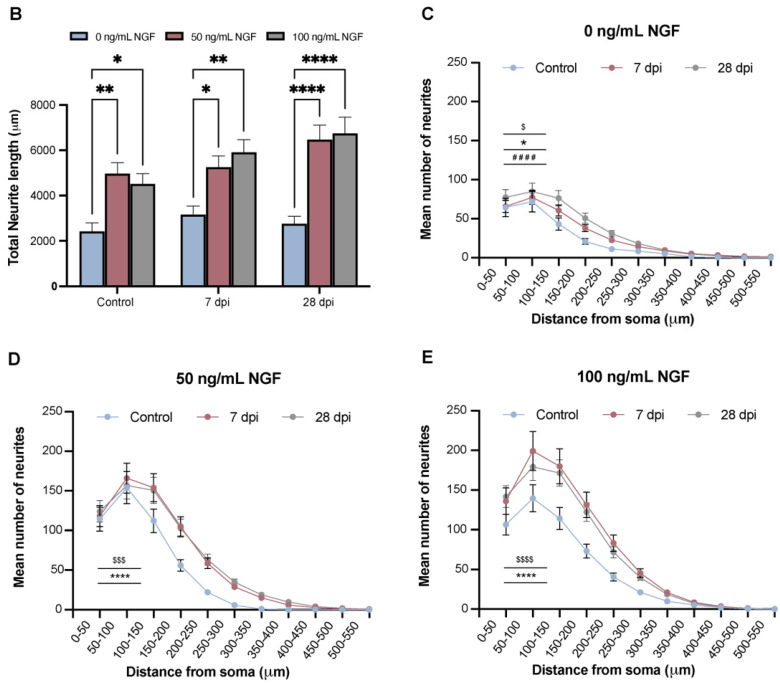
In vitro experiments using lumbosacral (L5-S1) DRG neurons from spinal intact (control) and SCT animals (7 and 28 dpi (days post-injury)) cultured for 22 h in distinct concentrations of NGF (0, 50 and 100 ng/mL). DRG neurons from control and SCT animals were immunostained against βIII- tubulin, and analysis showed that neurons emitted long and ramified neurites, which were more prominent in cells from SCT animals (**A**). Axonal growth was exacerbated by the presence of NGF in the culture medium. Scale bar: 100 µm. Total neurite length (**B**) of cultured DRG cells significantly increased in all experimental groups treated with either 50 ng/mL or 100 ng/mL of NGF, compared to animals treated with 0 ng/mL of NGF. Graphs represent mean ± SEM and *p* < 0.05 was considered statistically significant. * *p* ≤ 0.05; ** *p* ≤ 0.01; **** *p* ≤ 0.0001; two-way ANOVA followed by Newman–Keuls multiple comparison test. Correlation between mean number of branches and distance from the soma (**C**–**E**) indicated that branching was significantly upregulated by NGF dosage in SCT animals, compared to controls. Graphs represent mean ± SEM and *p* < 0.05 was considered statistically significant. (**C**) $ *p* ≤ 0.05 7 dpi vs. control animals; * *p* ≤ 0.05 28 dpi vs. control animals; #### *p* ≤ 0.0001 7 dpi vs. 28 dpi animals. (**D**) $$$ *p* ≤ 0.001 7 dpi vs. control animals; **** *p* ≤ 0.0001 28 dpi vs. control animals. (**E**) $$$$ *p* ≤ 0.0001 7 dpi vs. control animals; **** *p* ≤ 0.0001 28 dpi vs. control animals; two-way ANOVA followed by Tukey’s multiple comparison test.

Neurons adhered well to the substrate and emitted long neurites, easily discernible with βIII-tubulin immunostaining (Figure 6A). These neurites were particularly evident and long in DRG neurons obtained from SCT animals, even in the absence of NGF. Neurite length analysis showed that the presence of NGF in the medium induced significantly longer neurites in SCT animals (Figure 6B). Ramification of neurites was also prominent. Analysis of growing cells showed that branching was significantly and dose-dependently upregulated in cells obtained from SCT animals treated with NGF (Figure 6C–E). Altogether, these results show that DRG neurons obtained from SCT animals can emit longer and more ramified neurites than neurons from control animals. The length and ramification were positively correlated with NGF concentration in the medium, indicating this neurotrophin potentiates the SCT-induced activation of a growth program.

**Figure 7:**
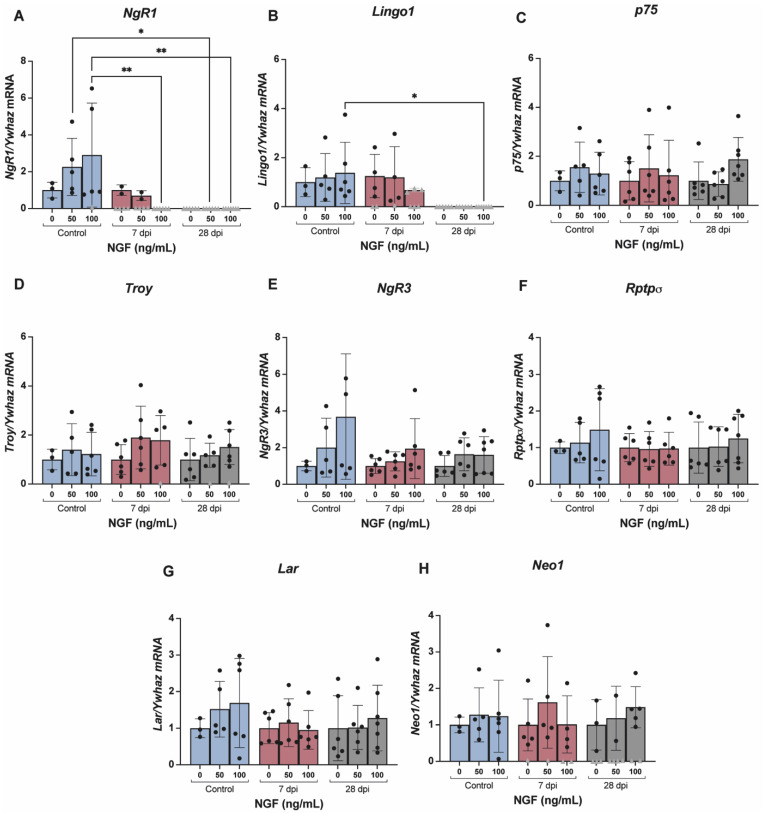
Levels of mRNA of myelin-associated inhibitors (MAIs) receptors *NgR1* (**A**), *Lingo1* (**B**), *p75* (**C**) and *Troy* (**D**), CSPGs receptors *NgR3* (**E**), *Rptpσ* (**F**) and *Lar* (**G**) and RGMa receptor *Neo1* (**H**) in cultured lumbosacral (L5-S1) DRG cells of spinal intact (control) and SCT animals. Graphs represent mean ± SD and *p* < 0.05 was considered statistically significant. mRNA levels of *NgR1* and *Lingo1* significantly decrease 28 dpi (days post-injury), independently of NGF concentration, as amplification was nondetected (represented in the graphs by grey triangles) in these groups. mRNA levels of the other receptors remained unaltered. * *p* ≤ 0.05; ** *p* ≤ 0.01; two-way ANOVA followed by Newman–Keuls multiple comparison test.

To investigate whether NGF would potentiate axonal growth by interfering with the expression of CSPGs, MAIs and repulsive guidance molecules receptors, RNA was extracted from cultured DRG cells from control and SCT animals (7 and 28 days post-injury) and reverse transcribed, and mRNA levels of *NgR1*, *Lingo1*, *p75*, *Troy*, *NgR3*, *Rptpσ*, *Lar* and *Neo1* were determined. In cultured neurons from SCT animals, exposure to NGF resulted in significant dose-dependent downregulation of the levels of *NgR1* and *Lingo1* (Figure 7A,B). Levels of the remaining receptors (Figure 7C–H) were not altered by SCT, nor by NGF exposure. It is important to mention that we were unable to determine expression by qPCR of some SCT samples (represented by grey triangles in Figure 7) for some of the studied receptors (*NgR1, Lingo1, Troy* and *Neo1*), although levels of the housekeeping gene *Ywhaz* were detected. As samples were run in triplicate and on two separate runs, it is feasible to assume that the lack of detection was not due to technical issues but rather reflects a lack of expression.

## 3. Discussion

Traumatic SCI is known to alter the composition of the spinal ECM at the injury site, where inhibitory cues that preclude axonal growth and regeneration of damaged tracts become accumulated. Although poorly investigated, there is also evidence that the ECM composition may be altered in segments distant from the injury location, and one can hypothesize that such changes likely contribute to maladaptive neuroplasticity, a matter investigated here.

In the present study, we used a model of complete spinal cord transection, which causes urinary dysfunction, a major concern for SCI patients [41,59]. SCI-induced urinary impairment is hypothesized as resulting from maladaptive reorganization of spinal circuits at the lumbosacral cord [60]. The chosen model has been previously validated [45] and, as before, we found that development of urinary dysfunction was accompanied by upregulation of GAP43 expression below the lesion. This protein is a marker of axonal sprouting [46] and was observed in lumbosacral DRG neurons and in the superficial laminae of the lumbosacral cord, where the central processes of bladder afferents terminate [48,49]. In SCT animals, these terminals have been previously shown to be peptidergic in nature, consistent with their bladder origin [42].

Axonal sprouting at the lumbosacral cord suggests changes in the spinal extracellular matrix, particularly in the superficial layers of the cord. To investigate this, spinal cord samples were analysed by Western blotting for expression of Phosphacan and Neurocan (CSPGs), Nogo-A, MAG and OMgp (MAIs) and RGMa. Surprisingly, we found an upregulation at 7 dpi of Neurocan, Phosphacan and Nogo-A, which coincided with increased GAP43 expression, with all proteins declining at 28 dpi. This is a puzzling observation, as those inhibitory proteins should have repressed axonal growth. However, the patterns of labelled structures did not coincide: immunolabelling showed that expression of GAP43 and Phosphacan and Nogo-A did not overlap, but rather coursed in parallel (Supplementary Figure S2G–I and Supplementary Figure S3G–I, respectively). Therefore, one could speculate that, as seen during development [61,62], the repulsive proteins act by directing the growing axons. It is well recognized that sprouting of peripheral afferents triggers the rearrangement of synaptic contacts at the lumbosacral cord and the final formation of new neuronal circuits. Hence, one can speculate that these circuits are restricted to the lumbosacral cord and operate without supraspinal input, leading to loss of voluntary control over bladder function and urinary incontinence [60,63]. Importantly, not all investigated inhibitory proteins were altered at the lumbosacral cord in response to thoracic spinal injury, suggesting that these dynamic changes are specific to undergoing neuroplastic rearrangements and not a generalized response.

The concurrent expression of GAP43 with Phosphacan, Neurocan and Nogo-A at the same time points and similar locations, but without colocalization, suggests that sensory afferents express receptors for those centrally produced inhibitory cues. To investigate this, the expression of key receptor components for CSPGs, MAIs and RGMa was analysed in lumbosacral DRG neurons collected from control and SCT animals. While mRNA levels of the RGMa receptor were not altered, in tandem with spinal protein levels, we found a time-dependent decrease in mRNA levels of CSPG- and MAI-receptors, which reached statistical significance at 28 dpi. Activation of these receptors with CSPGs or MAIs results in downstream intracellular activation of the RhoA/ROCK signalling pathway. When activated, ROCK catalyses actin depolymerization, growth cone collapse and arrest of axonal growth [64] via phosphorylation of several downstream targets, including MYPT1 [65]. In the present study, we found signs of axonal growth, as shown by upregulation of GAP43 expression and reduction in the expression of receptors for growth inhibitors in the DRG neurons. In line with these changes, activation of RhoA/ROCK, as evaluated by the levels of phosphorylated MYPT1, was minimal and not different between experimental groups. However, in addition to RhoA, regulation of neuronal growth by CSPGs, myelin-derived growth inhibitors and RGMa might also occur due to the activation of alternative pathways, e.g., inactivation of the pro-growth signalling cascade via Akt and glycogen synthase kinase-3 (GSK-3). When GSK-3 is blocked in rodent models of spinal cord transection and contusion, enhanced axonal growth of descending corticospinal and raphespinal tracts, as well as locomotor functional recovery was observed [66]. Accordingly, using three genetic mouse models with high, intermediate or no GSK3β activity in neurons, Liz and co-workers demonstrated that reduced levels or complete elimination of this enzyme in SCI animals promoted axon regeneration [67].

While our results indicate a time-dependent decrease in the peripheral expression of receptors for CSPGs and MAIs, associated with enhanced central axonal sprouting of sensory afferents, the underlying mechanisms remain to be elucidated. A likely candidate involved in these mechanisms is Nerve Growth Factor (NGF). NGF is a neurotrophin, essential for growth and survival of sensory and sympathetic neurons, playing a crucial role in both CNS recovery and dysfunction following SCI. NGF is upregulated following SCI [52,53,54] and, while in perilesional locations’ NGF increase could help to promote some regeneration and protection of surviving neuronal circuits [68,69,70], it has also been associated with severe SCI outcomes, such as autonomic dysreflexia, urinary dysfunction and pain [54,58,71,72,73], all of which have been linked to abnormal axonal sprouting [44,71]. Here, and in other studies [42,45], GAP43 upregulation has been found in the lumbosacral cord and DRG. As NGF regulates GAP43 [74], one can assume that in the present study, NGF levels could also be increased and would play a role in regulating the expression of receptors for CSPGs and MAIs. An indication in this direction was provided by our in vitro studies: lumbosacral DRG neurons were collected from control and SCT animals and cultured with different concentrations of NGF. The neurite outgrowth elicited by NGF was longer and more ramified in DRG neurons obtained from SCT animals, suggesting that these cells were primed by spinal trauma and their sprouting potential was enhanced by NGF exposure. Analysis of the expression of CSPG-, MAI- and RGMa-receptors in these cells showed a dose-dependent decrease in the levels of *NgR1* and *Lingo1*. As those receptors are part of multi-subunit complexes, reduction in the levels of a single subunit could compromise the activation of the whole receptor, blocking the inhibitory effects on axonal growth of Nogo-A, and possibly CSPGs. This is a novel mechanistic concept, as NGF is typically seen as a growth promoter by inducing the activation of pro-survival/pro-growth genes [75], rather than by reducing the expression of genes linked to inhibition of axonal growth. 

Interestingly, the effects of NGF seemed to be restricted to *NgR1* and *Lingo1*, as mRNA levels of the other studied receptors remained unaltered. The reasons for this are only speculative at present. However, it should be recalled that, as NGF overcomes the inhibitory effect of CSPGs in conditioned lesioned neurons via integrin-linked kinase (ILK) signalling [76], this might explain the lack of differences in mRNA levels of receptors associated with CSPGs (*NgR3*, *Lar*, *Rptpσ)* when NGF concentration in the medium increased. Moreover, while the importance of NGF in regulating axonal growth is indisputable, other potential molecules could also contribute. Several studies indicate that various growth factors co-participate in regulating axonal growth. Gavazzi et al. demonstrated that both NGF and Glial Cell line Derived Neurotrophic Factor (GDNF) are necessary for growth of sensory neurons [77]. Brain Derived Neurotrophic Factor (BDNF) and Neurotrophin 3 (NT-3) also participate in this process [42,78]. Neurite growth in vitro follows NGF-independent and NGF-dependent mechanisms, but is also influenced by other growth factors [79] and inflammatory mediators, including interleukin (IL)-1β and IL-6, the levels of which are upregulated at 7 dpi at spinal cord injury sites [80]. The present results are consistent with a major role played by NGF in the abnormal lumbosacral expansion of sensory bladder afferents. Several studies have shown that NGF manipulation, initiated at chronic stages after SCI, can improve bladder function in SCI rodents [57,58]. Those studies suggested that in SCI animals, NGF contributes to bladder dysfunction by upregulating the bladder expression of the ion channels P2X3, TRPA1 and TRPV1, and inducing hyperexcitability of capsaicin sensitive bladder afferents. Our results indicate that, in addition to published reports [57,58], the beneficial effects of NGF manipulation on bladder function could also include changes in the expression of genes involved in axon guidance, likely further contributing to harnessing maladaptive neuroplasticity.

## 4. Material and Methods

### 4.1. Animals

In-house bred adult female Wistar Han rats, derived from Charles River Laboratories (France) and weighing approximately 200–290 g, were maintained under a 12 h dark/light cycle, in a temperature and humidity-controlled environment with free access to food and water. Experimental procedures were carried out according to the European Communities Council Directive 2010/63/EU and local regulations (protocol ORBEA_56_2017/1218; 31 January 2019). All efforts were made to reduce the number of animals used, as well as animal stress and suffering.

### 4.2. Chemicals and Reagents

Spinal cord transection surgeries were performed under deep anaesthesia, induced by intraperitoneal injection of ketamine (75 mg/kg) and medetomidine (0.5 mg/kg) and reversed with atipamezole (1 mg/kg). For cystometries, animals received a subcutaneous injection of urethane (1.2 g/kg). For terminal procedures, rats were administered a lethal dose of sodium pentobarbital (65 mg/kg).

Primary and secondary antibodies used in Western blotting, immunohistochemistry and immunocytochemistry assays, as well as sources and dilutions, are listed in Table 1 and Table 2, respectively. For antibodies suitable for immunohistochemistry and Western blotting, the specificity was confirmed by immunolabelling of the injury site (Supplementary Figure S1) prior to Western blotting.

Chondroitinase ABC from *Proteus vulgaris* (C3667), Collagenase IV-S from *Clostridium histolyticum* (C1889) and Laminin from Engelbreth–Holm–Swarm murine sarcoma basement membrane (L2020) were purchased from Sigma-Aldrich, USA. DMEM (1X) + GlutaMAX™-I (31966-021), Fetal Bovine Serum (10082147) and Penicillin Streptomycin (15070-063) were purchased from Thermo Fisher Scientific, Oeiras, Portugal. Poly-L-Lysine (0413-SC) was bought from Quimigen, Alverca, Portugal. Nerve growth factor (mNGF, 2.5S) (G5141) was purchased from Promega. PowerUp™ SYBR™ Green Master Mix (A25777) was purchased from Alfagene, Carcavelos, Portugal. Primers were synthetized by STABvida, Caparica, Portugal.

### 4.3. Spinal Cord Transection

A complete spinal cord transection (SCT) at the level of the low thoracic spinal segments T8/T9 was used [45]. In SCT groups, T7 to T10 vertebrae were exposed and animals underwent a laminectomy at T8/T9 level. The spinal cord was sectioned with a scalpel and a sterile absorbable haemostatic sponge was placed between the sectioned ends of the cord. Muscle and skin were sutured in layers. SCT animals submitted to spinal transection were left to recover for 7 days or 28 days. Control animals were left spinal intact. To compensate for blood loss and poor oral water intake following SCT surgery, animals received a 1 mL subcutaneous injection of 5% saline–glucose. For discomfort and pain management, animals received oral tramadol (1 mL/kg) for an average period of 5 days post-surgery. Rats also received an oral suspension of enrofloxacin (5 mg/kg) for 7 days post-surgery. In SCT animals, bladders were manually emptied daily by abdominal compression and until automatic micturition developed, to avoid urinary retention and kidney damage.

### 4.4. Cystometries

At each time point, all animals received a subcutaneous bolus of urethane to induce deep anaesthesia. Bladders were exposed through a low midline abdominal incision and a 21-gauge needle connected to an infusion pump and to a pressure transducer was inserted into the bladder dome for saline infusion. Animals were placed on a heating pad and body temperature was kept at 37 °C. Fifteen minutes after needle insertion, saline infusion was initiated at a constant rate of 6 mL/h. Intraluminal pressure was recorded for 1 h and the urethra remained unobstructed so that infused saline could easily be expelled by bladder contractions. After cystometries, animals were euthanized with pentobarbital and the lesion site (T8/T9), lumbosacral spinal cord (L5-S1) and respective dorsal root ganglia (DRG) were collected for protein analysis (*n* = 6/group), qPCR (*n* = 7/group), immunohistochemistry (*n* = 4/group) and cell culture (*n* = 3–7/group). Cystometrograms were analysed and frequency, amplitude and peak and basal pressure of bladder reflex contractions were determined using LabScribe Software v4 (iWorx, World Precision Instruments, Friedberg, Germany). When amplitude of bladder contractions was less than 10 cm H_2_O, amplitude and frequency of bladder contractions was considered zero.

### 4.5. Western Blotting Analysis

For protein analysis, tissue was freshly collected and frozen at −80 °C. Lumbosacral spinal cord segments and lumbosacral DRG were homogenized using a MagNa Lyser Instrument (Roche Diagnostics, Mannheim, Germany) and 1.4 mm ceramic Beads (Qiagen, Hilden, Germany) in lysis buffer (Tris 50 nM, NaCl 150 nM, Triton X-100 0.5% (*v/v*), EDTA 1nM, pH 7.6) supplemented with cOmplete™ Protease Inhibitor Cocktail (Roche Diagnostics, Mannheim, Germany) and PhosSTOP Phosphatase Inhibitor Cocktail (Roche Diagnostics, Mannheim, Germany). Homogenates were sonicated and protein concentration was determined using the Bradford protein assay (Bio-Rad Laboratories, Hercules, CA, USA). For CSPGs detection, 30 μg of spinal tissue homogenates was treated with 0.3 U/mL chondroitinase ABC (ChABC) for 3 h at 37 °C [40]. Ch ABC activity was stopped by boiling the samples at 95°C for 15 min in 1 X gel-loading buffer (GLB). Proteins were electrophoresed on 8% SDS-polyacrylamide gels and transferred onto PVDF membranes using a Trans-Blot Turbo transfer system (Bio-Rad Laboratories, Hercules, CA, USA). Membranes were blocked in 5% (*w/w*) milk in Tris buffer saline with 0.1% (*w/w*) Tween-20 (TBS-T) and incubated overnight at 4 °C with specific primary antibodies (Table 1), diluted in 5% (*w/w*) bovine serum albumin (BSA) in TBST. Membranes were then incubated with HRP-conjugated secondary antibodies (Table 2) for 1 h at room temperature. Immunoblots were developed via chemiluminescence (SuperSignal™ West Pico PLUS Chemiluminescent Substrate, Thermo Fisher Scientific, Rockford, IL, USA). Digital images were obtained in a Chemidoc MP System Imager using an ImageLab 5.1 software (Bio-Rad Laboratories, Hercules, CA, USA). Protein levels were quantified by densitometry and normalized against GAPDH levels using Fiji software for Mac OS X.

### 4.6. Perfusion and Tissue Processing

For immunohistochemical tissue analysis, after cystometry, animals were submitted to immediate transcardiac fixative perfusion with calcium-free Tyrode’s solution (0.12 M NaCl, 5.4 mM KCl, 1.6 mM MgCl_2_·H_2_O, 0.4 mM MgSO_4_·H_2_O, 1.2 mM NaH_2_PO_4_·H_2_O, 5.5 mM glucose and 26.2 mM NaHCO_3_) followed by 4% paraformaldehyde (PFA) in 0.1 M phosphate buffer (PB). L5-S1 spinal segments and the spinal segment approximately 5 mm caudal and rostral to the injury site were collected, post-fixed overnight (ON) in 4% PFA, followed by immersion in 30% sucrose in 0.1 M PB with 0.1% sodium azide. Next, 20 μm of transverse serial sections of lumbosacral spinal cord tissue and 12 μm longitudinal sections of the injury site were obtained in a Leica cryostat, collected in Superfrost Plus slides and stored at −20 °C until further processing.

### 4.7. Immunohistochemistry Assays

Alternate slides were thawed and washed with phosphate-buffered saline (PBS) and PBS containing 0.3% Triton X-100 (PBST). Sections were blocked in PBST containing 10% normal horse serum (NHS) for 1 h. Primary antibodies (Table 1) were diluted in in PBST containing 2% NHS and sections incubated for 48 h at 4 °C. Sections were then washed with PBST and subsequently incubated for 1 h at room temperature with species-specific Alexa™ fluorochrome-labelled secondary antibodies (Table 3). After several washes with PBST, sections were slide covered and mounted with SlowFade Gold antifade mounting medium (Thermo Fisher Scientific, Rockford, IL, USA).

Representative images were obtained using a Zeiss microscope (Axioimager Z1, Zeiss Z1 from Zeiss, Oberkochen, Germany) using the Axiovision 4.8 software. For spinal GAP43 quantification, L5-S1 dorsal horns were captured at 10x using the same exposure settings for all sections. GAP43 expression in lamina I and II was quantified in at least 6 sections per segment. Briefly, the mean level and standard deviation of background immunofluorescence was obtained in the region of interest without visible GAP43 staining for each section analysed using ROI analysis (Fiji software for Mac OS X). The threshold level for GAP43 positive pixels was set at a value of 5 standard deviations above the mean background level. The mean percentage of GAP43 staining was then calculated by delimiting lamina I and II in each section and using ROI analysis [81].

### 4.8. RNA Extraction and cDNA Synthesis

Lumbosacral DRG neurons (L5-S1) were homogenized using a MagNa Lyser Instrument (Roche Diagnostics, Mannheim, Germany) and 1.4 mm ceramic beads (Qiagen, Hilden, Germany)). Total RNA was extracted from L5-S1 DRG tissue using the RNAqueous^®^-Micro kit (Applied Biosystems, Bedford, MA, USA). An aliquot of each RNA sample was run on a denaturing agarose gel to assess integrity. Intact RNA was then quantified by NanoDrop 2000 (Thermo Scientific, Waltham, MA, USA), followed by treatment with DNase. DNase-treated RNA (500 ng) was reverse transcribed using the NZY M-MuLV First-Strand cDNA Synthesis Kit (NZYtech, Lisbon, Portugal), according to the manufacturer’s instructions. cDNA was diluted and stored at −20 °C for later use.

### 4.9. Quantitative Real-Time PCR (qPCR)

Relative gene expression levels were measured by real-time qPCR using the StepOnePlus Real-time PCR system (Applied Biosystems, Bedford, MA, USA). Reaction mixes were prepared using 7 µL of PowerUp™ SYBR™ Green Master Mix (Applied Biosystems, Bedford, MA, USA), 1 µL of primer mix (forward + reverse primers in a final concentration of 10 µM), 5 µL of Milli-Q H_2_O and 1 µL of cDNA sample, to a final volume of 14 µL. Each sample was run in triplicate. The amplification protocol consisted of forty cycles with denaturation at 95 °C for 15 s, followed by annealing at predetermined temperatures that varied from 58–60 °C, for 30 s, and extension at 72 °C for 30 s.

Target gene expression was normalized against the expression of the endogenous ATP Synthase F1 Subunit Beta (*Atp5f1b*) gene in in vivo samples and against Tyrosine 3-Monooxygenase/Tryptophan 5-Monooxygenase Activation Protein Zeta (*Ywhaz*) in in vitro samples. Primer sequences and respective Tm are listed in Table 3.

### 4.10. Primary DRG Cell Culture

L5-S1 DRG were collected from control and SCT animals (7 and 28 days following injury) (*n* = 3–7 per group) and placed in Dulbecco’s modified Eagle’s medium (DMEM)-Glutamax with 10% foetal bovine serum (FBS) and 1% penicillin/streptomycin. Nodules underwent enzymatic digestion with 0.125% collagenase IV-S for 2 h at 37 °C, followed by mechanical dissociation with glass Pasteur pipettes of increasingly small diameters. The single-cell solution was purified and resuspended in DMEM-Glutamax with 1% penicillin/streptomycin, 2% B27 and 0, 50 or 100 ng/mL of NGF. Cells were plated onto poly-L-lysine and laminin-coated coverslips or wells for immunocytochemistry and qPCR assays, respectively. Cells were maintained at 37°C in a humidified 5% CO_2_ atmosphere for 22 h. For immunocytochemistry assays, coverslips were washed with ice-cold PBS and fixed with 4% PFA for 15 min. After several washes with PBST for cell membrane permeabilization, cells were blocked with 5% NHS in PBST for 1 h and then incubated overnight with anti-βIII-tubulin in 2% NHS in PBST (Table 1), at 4° C. Cells were then washed several times and incubated for 1 h at room temperature with a species-specific Alexa Fluorochrome-labelled antibody (Table 2) in 2% NHS in PBST. After washing, coverslips were mounted with SlowFade Gold antifade mounting medium (Thermo Fisher Scientific, USA). Quantification of neurite branching (Scholl analysis) and total neurite length analysis were performed with SYnapse Detector (SynD) [82], using MATLAB R2021a. 

From other coated wells, total RNA was extracted from DRG neurons cultured using RNAqueous-Micro kit (Applied Biosystems). The same procedure was followed as previously described for in vivo RNA extraction from DRG. 

### 4.11. Data Analysis

Data from in vivo Western blotting, immunohistochemistry and in vivo qPCR assays were statistically analysed using one-way ANOVA followed by Tukey’s multiple comparison post hoc test using GraphPad Prism 9 software. Results are represented as mean ± SD and *p* < 0.05 was considered statistically significant. Cell culture data to obtain Scholl analysis and neurite length were analysed in MATLAB using SYnapse Detector (SynD) [82], a synapse and neurite detection software program. SynD can quantify neurite branching by measuring the number of cellular processes crossing concentric circles placed around the cell body and measure neurite lengths. Statistical analysis was performed in GraphPad Prism 9 software. For total length of neurite extension, neurite branching and for each distance interval from the nucleus, as well as in vitro qPCR analysis, two-way ANOVA followed by the Student–Newman–Keuls Method post hoc test was used. In these instances, values are presented as mean ± SEM. *p* < 0.05 was considered statistically significant.

## 5. Conclusions

Traumatic SCI is a catastrophic event, with life-changing consequences arising from the lack of regeneration of neuronal tracts. At the lesion site, the extracellular environment becomes highly repulsive, which averts neuronal rewiring. The present study showed that a similar phenomenon occurred in spinal segments distant from the injury site. Such changes could lead to maladaptive neuroplasticity, resulting in typical complications such as urinary dysfunction. We showed that urinary dysfunction arising after complete thoracic spinal cord transection was accompanied by central sprouting of sensory afferents at the lumbosacral cord, in parallel to upregulation of inhibitory cues (Phosphacan, Neurocan, Nogo-A). Expression at the DRG of receptors for Phosphacan, Neurocan and Nogo-A was time-dependently downregulated, thus enhancing afferent fibre sprouting. Such changes could, at least partially, be mediated by peripheral NGF, as NGF downregulated inhibitory factor receptor subunits in cultured DRG neurons. Altogether, our results confirm the occurrence and importance of molecular mechanisms operating far from the injury site than can be accounted for maladaptive neuroplastic events. Moreover, data also support that NGF manipulation should be further explored as a potential therapeutic tool to harness neuroplasticity and control SCI-induced urinary impairment.

## Figures and Tables

**Table 1:** Primary antibodies used in Western Blotting, Immunohistochemistry and Immunocitochemistry assays.

Primary Antibody Name	Catalogue #	Company	MW	Source	Dilution for WB	Dilution for IHC/ICC
Nogo-A	11C7	Kindly gifted by Prof. Martin Schwab	180 kDa	Mouse	1:1000	1:1000
Phosphacan	MAB5210	Merck Millipore	180, 190 and 250 kDa	Mouse	1:1000	1:1000
Neurocan	MAB5212	Merck Millipore	245, 160, 130 and 90 kDa	Mouse	1:1000	1:1000
Omgp	ab109746	Abcam	28 kDa	Rabbit	1:5000	-
Mag	ab203060	Abcam	69 kDa	Rabbit	1:1000	-
Rgma	28045	IBL Japan	28 kDa	Rabbit	1:500	-
GAP43	ab16053	Abcam	43–48 kDa	Rabbit	1:2000	1:2000
GAPDH	ab8245	Abcam	37 kDa	Mouse	1:10,000	-
βIII tubulin	302302	Synaptic Systems	55 kDa	Rabbit	1:1000	1:1000
MYPT1	#2634	Cell Signalling	140 kDa	Rabbit	1:1000	-
Phospho-MYPT1 (Thr696)	#5163	Cell Signalling	140 kDa	Rabbit	1:1000	-

**Table 2:** Secondary antibodies used in Western Blotting (WB), Immunohistochemistry (IHC) and Immunocitochemistry (ICC) assays.

Secondary Antibody Name	Catalogue #	Company	Source	Dilution for WB	Dilution for IHC/ICC
Alexa Fluor™ 568 Donkey Anti-Rabbit IgG	A10042	Invitrogen	Donkey	-	1:1000
Alexa Fluor™ 488 Donkey Anti-Rabbit IgG	A21206	Invitrogen	Donkey	-	1:1000
Alexa Fluor™ 568 Donkey Anti-Mouse IgG	A10037	Invitrogen	Donkey	-	1:1000
Goat Anti-Rabbit IgG (HRP)	ab6721	Abcam	Goat	1:10,000	1:1000
Goat Anti-Mouse IgG (HRP)	ab205719	Abcam	Goat	1:10,000	-

**Table 3:** Primer (forward and reverse) sequences, annealing temperatures and NCBI accession numbers.

Gene Name	Gene Bank Accession Number	Primer Sequence (5′→3′)	Annealing Temperature (°C)
*Atp5f1b*	NM_134364.1	**Forward:** TGC TAT TGC TGA GTT GGG CA **Reverse:** GGA GAT GGT CAT AGT CAC CTGC	60
*Gap43*	NM_017195.3	**Forward:** ACC ACT GAT AAC TCG CCG TC **Reverse:** TGG CTT CAT CTA CAG CTT CTT TCT	60
*Lar (Ptprf)*	NM_019249.1	**Forward:** CTC CCA GCG CTT TGA GGT AA **Reverse:** GAG CCT TCT CCA CCA CCT TC	59
*Lingo1*	NM_001100722.1	**Forward:** CTC CTG AGC TCC TGC CCT A **Reverse:** CTC TCG CTA CCT GCA TCT C	60
*Neo1*	XM_008766432.1	**Forward:** GTT GCT AGG GAG CGT GTT GA **Reverse:** GTA GGC CAC CAC TCG GAA AC	60
*NgR1 (Rtn4r)*	NM_053613.1	**Forward:** CGA AGA TGA AGA GGG CGT CC **Reverse:** TAG TTG CTC CAG GAG GGT CA	60
*NgR3 (Rtn4rl1)*	NM_181377.3	**Forward:** GGG ATT TGA ATC TGG ACC CCA **Reverse:** CAA TTC CAC ACA GCA CCC TTT G	59
*P75^NTR^ (Ngfr)*	NM_012610.2	**Forward:** TGA CTC TCC ATT TGG GAC TTT **Reverse:** TAC GGG TGC GGT TCT TTC	60
*RPTPRσ (Ptprs)*	NM_019140.2	**Forward:** CAG ACG CCA GGT ATG CTC AG **Reverse:** ATA GGC GAT GAC ATT GGC GT	60
*Troy (Tnfrsf19)*	NM_001044229.2	**Forward:** TCT GCA AAC AGT GTG GAC CT **Reverse:** TCT TCC GGT AAA ATC CTG GCA	58
*Ywhaz*	NM_013011.4	**Forward:** ACC CAC TCC GGA CAC AGA ATA **Reverse:** TCG AGA CGA CCC TCC AAG AT	60

## Data Availability

All data presented in this study are available under request by contacting the corresponding author.

## Supplementary Materials

The supporting information can be downloaded at: https://www.mdpi.com/article/10.3390/ijms23158667/s1.
