# Supplementary material for: Development of Neurogenic Detrusor Overactivity after Thoracic Spinal Cord Injury Is Accompanied by Time-Dependent Changes in Lumbosacral Expression of Axonal Growth Regulators
Source: Int J Mol Sci. 2022 Aug 4;23(15):8667. doi: 10.3390/ijms23158667 (PMC9368817; doi:10.3390/ijms23158667)
Supplement: Supplementary file 1 [file ijms-23-08667-s001.zip › Chambel et al Legendas July 14th (REVISED by all).pdf]

Supplementary Figure S1. Immunodetection of time-dependent expression of growth inhibitory molecules Nogo-A (A-D) and Phosphacan (E-H), as well as neural sprouting marker GAP43, in transverse sections of the lesion site of thoracic spinal segments T8/T9 (±1 cm from the lesion epicenter) of SCT (7 and 28 dpi (days post-injury)) animals. Fluorescent immunohistochemistry images show the extent of the injuries after thoracic spinal cord transection 7 and 28 dpi. The expression of repulsive molecules in the periphery of the injury epicenter is evident (A, B, E, F). The boxed areas in * are enlarged on panels (C,D and G, H). Scale A, B, E and F = 500 µm; Scale C, D, G, H = 100 µm.

Supplementary Figure S2. Time-dependent expression of Phosphacan in lumbosacral spinal cord segments of spinal intact (control) and SCT animals (7 and 28 dpi (days post-injury)). Panoramic view of L6 spinal cord segments immunoreacted for Phosphacan in control (A), 7 dpi (B) and 28 dpi (C) animals. Phosphacan immunoreaction in the dorsal horn (dh) of L6 spinal segments of control (D) and 7 dpi (E) and 28 dpi (F) shows an increase in Phosphacan expression 7 dpi throughout the horn, especially in the superficial laminae. Magnification of the superficial lamina of the dorsal horn (represented by boxed in *) in control (G), 7 dpi (H) and 28 dpi (I) shows some co-localization with the sprouting marker GAP43, especially at 7 dpi. Phosphacan expression in the dorsal commissure (dc) (J-L) and the ventral horn (vh) (M-O) of lumbosacral segments also show an increase in Phosphacan immunofluorescence in these areas. Scale A, B and C = 200 µm; Scale D-O = 50 µm.

Supplementary Figure S3. Time-dependent expression of Nogo-A in lumbosacral spinal cord segments of spinal intact (control) and SCT animals (7 and 28 dpi (days post-injury)). Panoramic view of L6 spinal cord segments immunoreacted for Nogo-A in control (A), 7 dpi (B) and 28 dpi (C) animals. Nogo-A immunoreaction in the dorsal horn (dh) of L6 spinal segments of control (D) and 7 dpi (E) and 28 dpi (F) shows a decrease in Nogo-A expression 28 dpi throughout the horn.

Magnification of the superficial lamina of the dorsal horn (represented by boxed in *) in control (G), 7 dpi (H) and 28 dpi (I) shows some co-localization with sprouting marker GAP43 at 7 dpi, disappearing 28 dpi. Nogo-A expression in the dorsal commissure (dc) (J-L) and the ventral horn (vh) (M-O) of lumbosacral segments also show an increase in Nogo-A immunofluorescence in these areas, especially in motoneurons of the ventral horn. Scale A, B and C = 200 µm; Scale D-O = 50 µm.
