# Supplementary figures and images for: Development of Neurogenic Detrusor Overactivity after Thoracic Spinal Cord Injury Is Accompanied by Time-Dependent Changes in Lumbosacral Expression of Axonal Growth Regulators
Source: Int J Mol Sci. 2022 Aug 4;23(15):8667. doi: 10.3390/ijms23158667 (PMC9368817; doi:10.3390/ijms23158667)

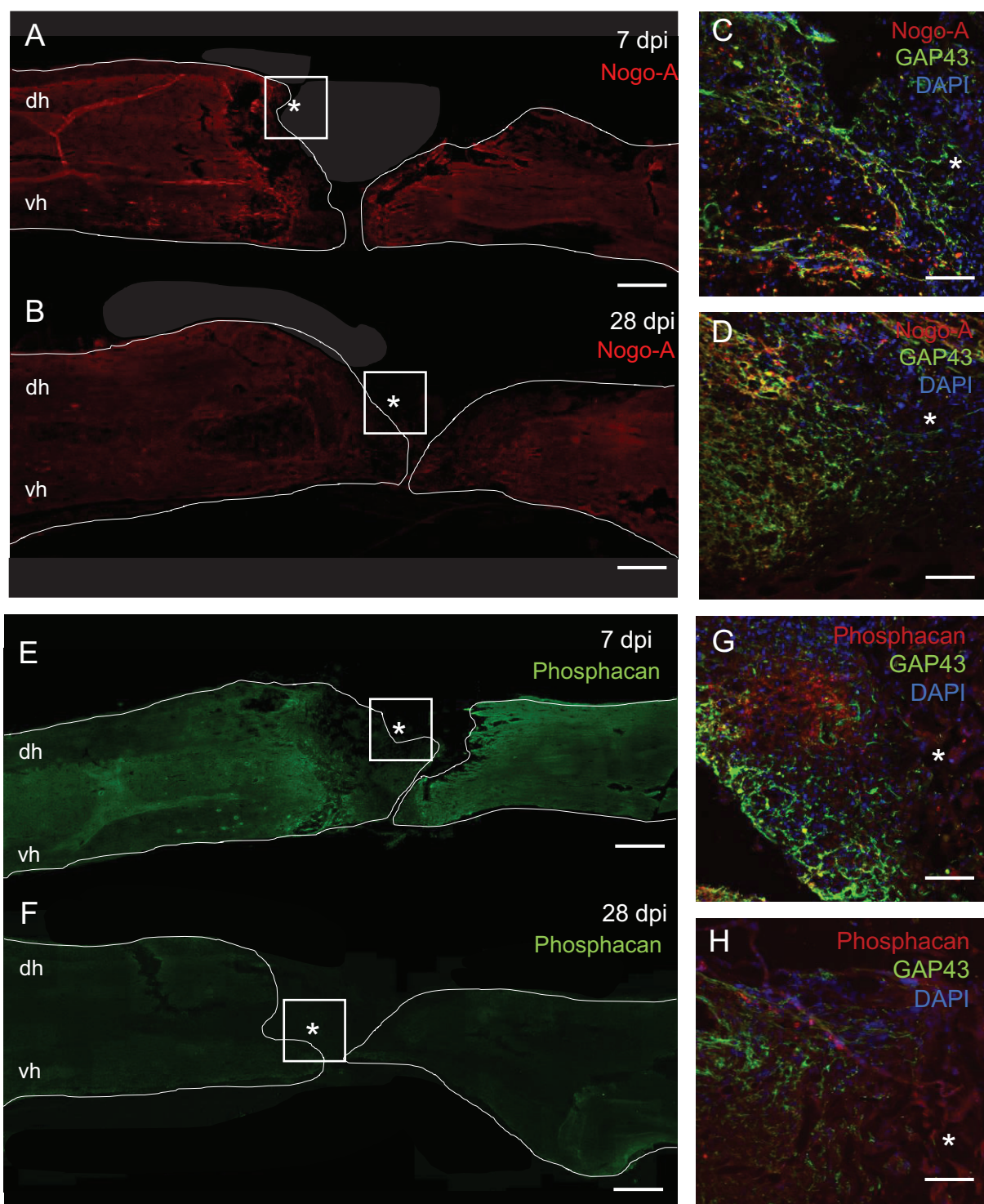

**Supplementary Figure S1**

Supplement: Supplementary file 1 [file ijms-23-08667-s001.zip › Supplementary figure S1 28th June - IHC Lesion sites Repulsive cues.pdf]

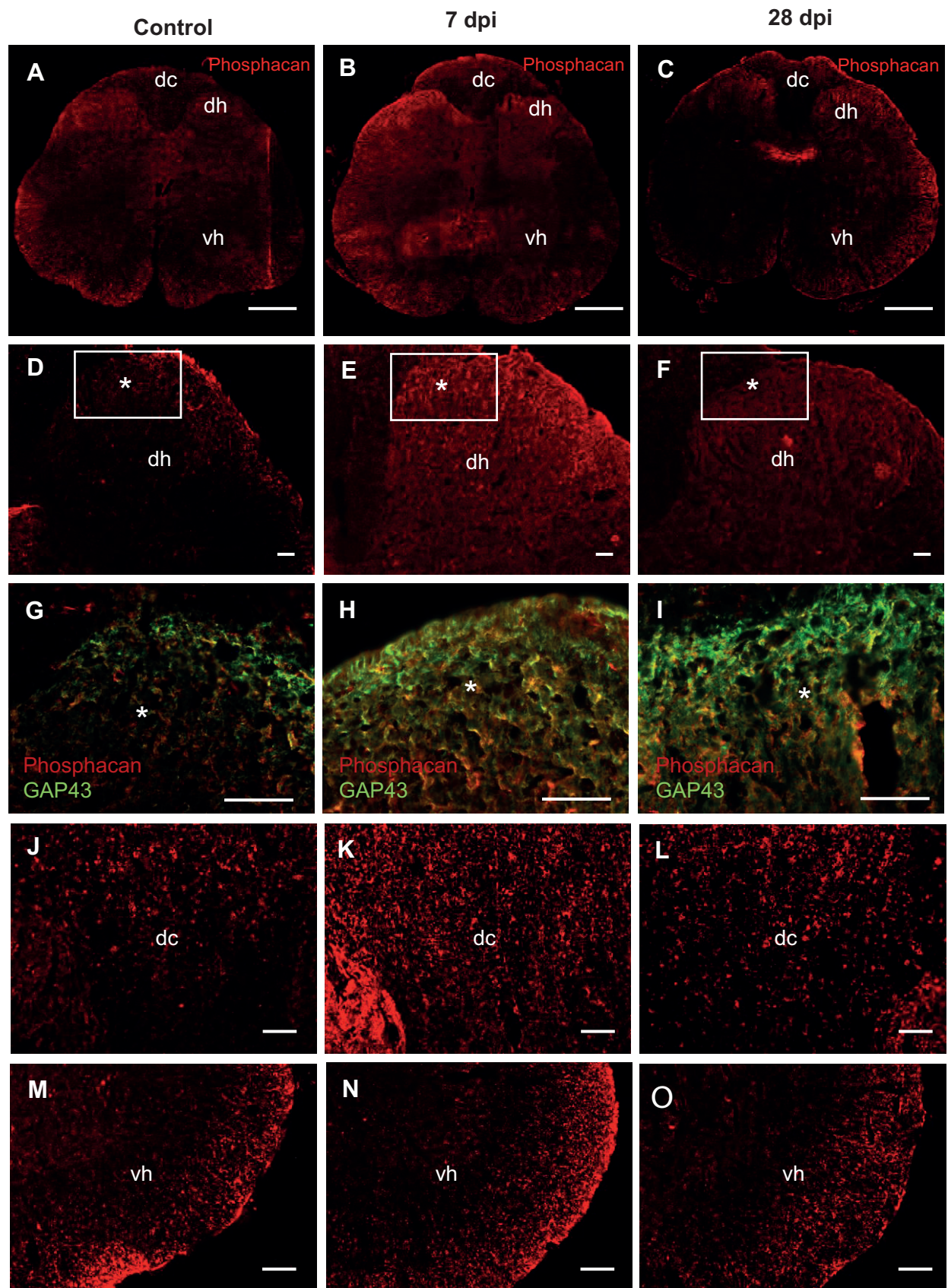

**Supplementary Figure S2**

Supplement: Supplementary file 1 [file ijms-23-08667-s001.zip › Supplementary figure S2 28th June - IHC SC Phosphacan.pdf]

7 dpi

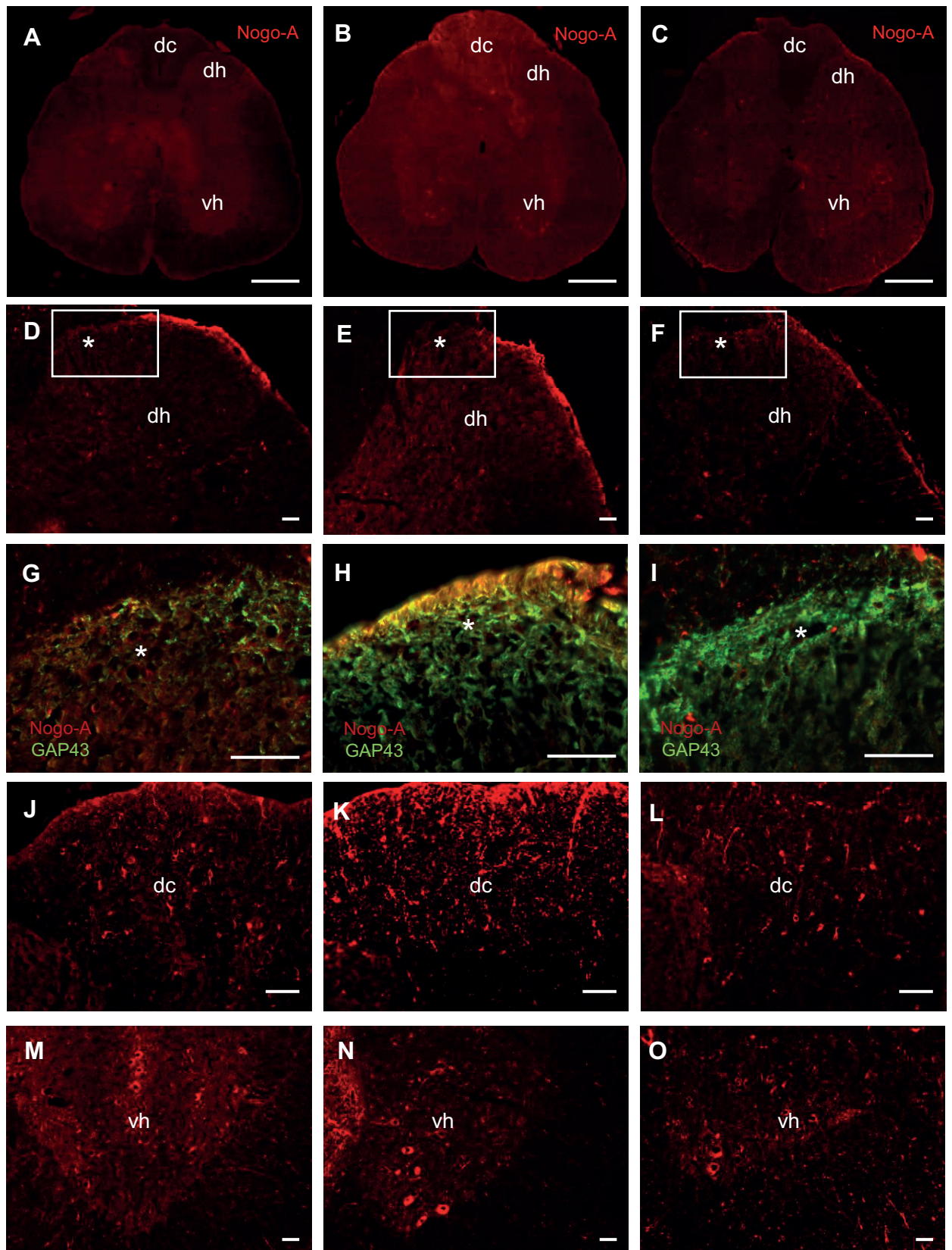

Supplementary Figure S3

Supplement: Supplementary file 1 [file ijms-23-08667-s001.zip › Supplementary figure S3 28th June - IHC SC Nogo-A.pdf]
